# 2-(4-Methyl­phen­yl)-1-phenyl­sulfonyl-3-nitro-1,2-dihydro­quinoline

**DOI:** 10.1107/S1600536811030455

**Published:** 2011-08-02

**Authors:** J. Kanchanadevi, G. Anbalagan, V. Saravanan, A. K. Mohanakrishnan, V. Manivannan

**Affiliations:** a Department of Physics, Velammal Institute of Technology, Panchetty, Chennai 601 204, India; b Department of Physics, Presidency College (Autonomous), Chennai 600 005, India; c Department of Organic Chemistry, University of Madras, Guindy campus, Chennai 600 025, India; d Department of Research and Development, PRIST University, Vallam, Thanjavur - 613 403, Tamil Nadu, India

## Abstract

In the title compound, C_22_H_18_N_2_O_4_S, the dihedral angle between the phenyl­sulfonyl ring and the methyl­phenyl ring is 67.78 (7)°. In the crystal, mol­ecules are linked by weak inter­molecular C—H⋯O inter­actions into a zigzag chain along the [101] direction.

## Related literature

For the biological activity of quinoline derivatives, see: Franck *et al.* (2004 ▶); Zouhiri *et al.* (2005 ▶); Paul *et al.* (1969 ▶). For a related structure, see: Xu *et al.* (2011 ▶).
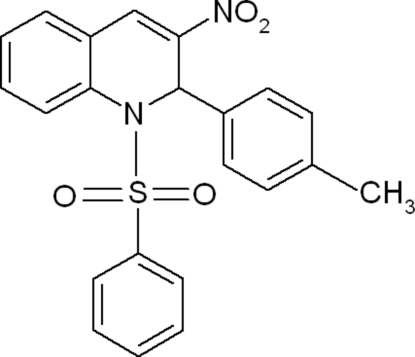


## Experimental

### 

#### Crystal data

C_22_H_18_N_2_O_4_S
*M* *_r_* = 406.44
Monoclinic, 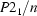

*a* = 9.7349 (5) Å
*b* = 17.0241 (9) Å
*c* = 12.1068 (6) Å
β = 90.240 (2)°
*V* = 2006.42 (18) Å^3^
*Z* = 4
Mo *K*α radiation
μ = 0.19 mm^−1^
*T* = 295 K
0.35 × 0.30 × 0.25 mm

#### Data collection

Bruker Kappa APEXII CCD diffractometer
Absorption correction: multi-scan (*SADABS*; Sheldrick, 1996 ▶) *T*_min_ = 0.945, *T*_max_ = 0.955
26473 measured reflections
5485 independent reflections
3224 reflections with *I* > 2σ(*I*)
*R*_int_ = 0.028

#### Refinement

*R*[*F* ^2^ > 2σ(*F* ^2^)] = 0.048
*wR*(*F* ^2^) = 0.151
*S* = 1.03
5485 reflections
263 parameters
H-atom parameters constrained
Δρ_max_ = 0.21 e Å^−3^
Δρ_min_ = −0.23 e Å^−3^

### 

Data collection: *APEX2* (Bruker, 2003 ▶); cell refinement: *SAINT* (Bruker, 2003 ▶); data reduction: *SAINT*; program(s) used to solve structure: *SHELXS97* (Sheldrick, 2008 ▶); program(s) used to refine structure: *SHELXL97* (Sheldrick, 2008 ▶); molecular graphics: *PLATON* (Spek, 2009 ▶); software used to prepare material for publication: *SHELXL97*.

## Supplementary Material

Crystal structure: contains datablock(s) global. DOI: 10.1107/S1600536811030455/is2758sup1.cif

Supplementary material file. DOI: 10.1107/S1600536811030455/is2758globalsup2.cml

Additional supplementary materials:  crystallographic information; 3D view; checkCIF report

## Figures and Tables

**Table 1:** Hydrogen-bond geometry (Å, °)

*D*—H⋯*A*	*D*—H	H⋯*A*	*D*⋯*A*	*D*—H⋯*A*
C4—H4⋯O4^i^	0.93	2.60	3.418 (4)	148

Symmetry code: (i) 
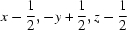
.

**Fig. 1.**
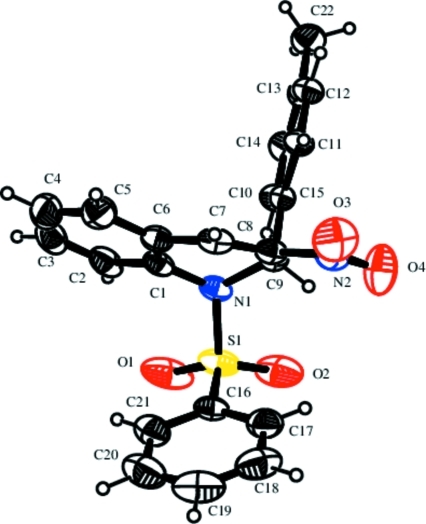
The molecular structure of the title compound, with atom labels and 30% probability displacement ellipsoids for non-H atoms.

**Fig. 2.**
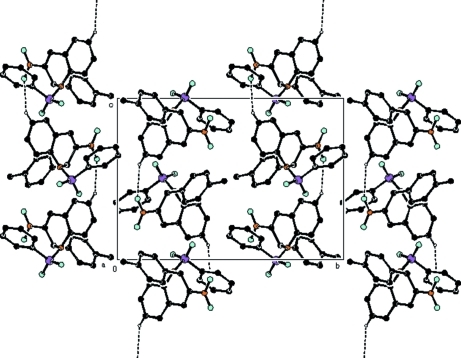
The packing of the title compound, viewed down the a axis. The C—H···O hydrogen bonds are shown as dashed lines.

## supplementary crystallographic information

### Comment

The quinoline and its derivatives have received much scientific attention during recent years, because of their wide spectrum of pharmacological activities (Franck *et al.*, 2004; Zouhiri *et al.*, 2005). In addition, the nitroquinoline derivatives possess a potent mutagenic, carcinogenic and carcinostatic agent (Paul *et al.*, 1969).

The geometric parameters of the title molecule (Fig. 1) agree well with a reported similar structure (Xu *et al.*, 2011). The phenylsulfonly ring and the methylphenyl ring are oriented at an angle of 67.78 (7)°. The sum of bond angles around N1 [352.34 (13)°] and N2 [359.95 (2)°] indicates the *sp^2^* hybridization state of atoms N1 and N2 in the molecule. The crystal packing is controlled by a weak intermolecular C—H···O interaction.

### Experimental

To a solution of N-(2-formylphenyl) benzenesulfonamide (0.50 g, 1.91 mmol) in dry benzene (20 ml), DABACO (0.10 g, 0.95 mmol) and 1-methyl-4-(2-nitrovinyl)benzene (0.41 g, 2.29 mmol) were added. The reaction mixture was stirred at reflux condition for 24 hrs under N_2_ atmosphere. The reaction mass was quenched with ice water (50 ml), extracted with chloroform (3 × 10 ml) and dried (Na_2_SO_4_). The solvent was removed under reduced pressure. Then the column chromatographic purification of crude product afforded pure dihydro nitroquinoline 18 as pale yellow solid with a yield of 82% and a melting point of 451 K.

### Refinement

H atoms were positioned geometrically and refined using riding model with C—H = 0.93 Å and *U*_iso_(H) = 1.2*U*_eq_(C) for aromatic C—H, C—H = 0.98 Å and *U*_iso_(H) = 1.2*U*_eq_(C) for C—H, C—H = 0.96 Å and *U*_iso_(H) = 1.5*U*_eq_(C) for CH_3_.

### Crystal data

**Table:**

C_22_H_18_N_2_O_4_S	*F*(000) = 848
*M**_r_* = 406.44	*D*_x_ = 1.346 Mg m^−^^3^
Monoclinic, *P*2_1_/*n*	Mo *K*α radiation, λ = 0.71073 Å
Hall symbol: -P 2yn	Cell parameters from 5485 reflections
*a* = 9.7349 (5) Å	θ = 2.1–29.4°
*b* = 17.0241 (9) Å	µ = 0.19 mm^−^^1^
*c* = 12.1068 (6) Å	*T* = 295 K
β = 90.240 (2)°	Block, pale yellow
*V* = 2006.42 (18) Å^3^	0.35 × 0.30 × 0.25 mm
*Z* = 4	

### Data collection

**Table:**

Bruker Kappa APEXII CCD diffractometer	5485 independent reflections
Radiation source: fine-focus sealed tube	3224 reflections with *I* > 2σ(*I*)
graphite	*R*_int_ = 0.028
Detector resolution: 0 pixels mm^-1^	θ_max_ = 29.3°, θ_min_ = 2.1°
ω and φ scans	*h* = −13→7
Absorption correction: multi-scan (*SADABS*; Sheldrick, 1996)	*k* = −23→23
*T*_min_ = 0.945, *T*_max_ = 0.955	*l* = −14→16
26473 measured reflections	

### Refinement

**Table:**

Refinement on *F*^2^	Primary atom site location: structure-invariant direct methods
Least-squares matrix: full	Secondary atom site location: difference Fourier map
*R*[*F*^2^ > 2σ(*F*^2^)] = 0.048	Hydrogen site location: inferred from neighbouring sites
*wR*(*F*^2^) = 0.151	H-atom parameters constrained
*S* = 1.03	*w* = 1/[σ^2^(*F*_o_^2^) + (0.066*P*)^2^ + 0.3469*P*] where *P* = (*F*_o_^2^ + 2*F*_c_^2^)/3
5485 reflections	(Δ/σ)_max_ < 0.001
263 parameters	Δρ_max_ = 0.21 e Å^−^^3^
0 restraints	Δρ_min_ = −0.23 e Å^−^^3^

### Fractional atomic coordinates and isotropic or equivalent isotropic displacement parameters (Å^2^)

**Table:**

	*x*	*y*	*z*	*U*_iso_*/*U*_eq_	
C1	0.19131 (16)	0.20602 (10)	0.83358 (16)	0.0572 (4)	
C2	0.1021 (2)	0.14330 (13)	0.8465 (2)	0.0782 (6)	
H2	0.0747	0.1278	0.9168	0.094*	
C3	0.0545 (2)	0.10423 (15)	0.7552 (3)	0.0984 (9)	
H3	−0.0059	0.0624	0.7640	0.118*	
C4	0.0945 (3)	0.12577 (17)	0.6509 (3)	0.1007 (9)	
H4	0.0622	0.0981	0.5898	0.121*	
C5	0.1819 (2)	0.18787 (15)	0.6368 (2)	0.0822 (6)	
H5	0.2078	0.2028	0.5660	0.099*	
C6	0.23211 (18)	0.22870 (11)	0.72767 (15)	0.0580 (4)	
C7	0.32475 (19)	0.29435 (10)	0.71606 (15)	0.0583 (5)	
H7	0.3352	0.3188	0.6479	0.070*	
C8	0.39412 (17)	0.31926 (10)	0.80240 (15)	0.0551 (4)	
C9	0.38459 (17)	0.27927 (10)	0.91304 (14)	0.0534 (4)	
H9	0.4006	0.3186	0.9707	0.064*	
C10	0.48831 (16)	0.21338 (10)	0.92830 (13)	0.0483 (4)	
C11	0.59619 (17)	0.20168 (11)	0.85758 (14)	0.0562 (4)	
H11	0.6054	0.2337	0.7958	0.067*	
C12	0.69139 (18)	0.14296 (12)	0.87689 (16)	0.0639 (5)	
H12	0.7646	0.1368	0.8285	0.077*	
C13	0.68006 (18)	0.09341 (11)	0.96638 (15)	0.0588 (4)	
C14	0.5720 (2)	0.10586 (12)	1.03651 (17)	0.0709 (5)	
H14	0.5621	0.0734	1.0977	0.085*	
C15	0.4780 (2)	0.16477 (12)	1.01928 (16)	0.0671 (5)	
H15	0.4068	0.1720	1.0693	0.080*	
C16	0.06971 (19)	0.37453 (12)	0.92595 (15)	0.0646 (5)	
C17	0.1392 (2)	0.44441 (15)	0.9176 (2)	0.0860 (7)	
H17	0.2226	0.4515	0.9540	0.103*	
C18	0.0829 (4)	0.50453 (16)	0.8536 (3)	0.1066 (9)	
H18	0.1291	0.5521	0.8465	0.128*	
C19	−0.0370 (4)	0.4936 (2)	0.8029 (3)	0.1156 (11)	
H19	−0.0737	0.5344	0.7610	0.139*	
C20	−0.1077 (3)	0.4253 (2)	0.8102 (2)	0.1116 (10)	
H20	−0.1919	0.4195	0.7746	0.134*	
C21	−0.0529 (2)	0.36413 (16)	0.8715 (2)	0.0861 (7)	
H21	−0.0988	0.3163	0.8757	0.103*	
C22	0.7804 (2)	0.02762 (14)	0.9861 (2)	0.0873 (7)	
H22A	0.8684	0.0491	1.0056	0.131*	
H22B	0.7889	−0.0032	0.9201	0.131*	
H22C	0.7480	−0.0050	1.0451	0.131*	
N1	0.24454 (14)	0.24821 (9)	0.92602 (12)	0.0584 (4)	
N2	0.48784 (19)	0.38510 (10)	0.79296 (19)	0.0773 (5)	
O1	0.02787 (19)	0.24655 (12)	1.03159 (17)	0.1265 (8)	
O2	0.2199 (2)	0.33166 (15)	1.09148 (12)	0.1231 (8)	
O3	0.5112 (2)	0.41204 (10)	0.70211 (16)	0.1096 (6)	
O4	0.5403 (2)	0.41024 (12)	0.8764 (2)	0.1219 (7)	
S1	0.13716 (6)	0.29822 (4)	1.00593 (4)	0.0809 (2)	

### Atomic displacement parameters (Å^2^)

**Table:**

	*U*^11^	*U*^22^	*U*^33^	*U*^12^	*U*^13^	*U*^23^
C1	0.0441 (8)	0.0530 (10)	0.0747 (12)	0.0120 (7)	0.0062 (8)	0.0164 (9)
C2	0.0526 (10)	0.0659 (13)	0.1161 (18)	0.0055 (9)	0.0055 (11)	0.0266 (13)
C3	0.0614 (13)	0.0625 (14)	0.171 (3)	0.0024 (11)	−0.0212 (17)	0.0103 (18)
C4	0.0879 (17)	0.0826 (18)	0.131 (3)	0.0143 (14)	−0.0380 (17)	−0.0154 (17)
C5	0.0858 (15)	0.0804 (16)	0.0801 (15)	0.0196 (13)	−0.0177 (12)	−0.0004 (12)
C6	0.0548 (9)	0.0569 (10)	0.0622 (11)	0.0153 (8)	0.0004 (8)	0.0097 (9)
C7	0.0646 (10)	0.0564 (10)	0.0541 (10)	0.0179 (8)	0.0164 (8)	0.0176 (8)
C8	0.0548 (9)	0.0469 (9)	0.0638 (11)	0.0081 (7)	0.0159 (8)	0.0115 (8)
C9	0.0556 (9)	0.0530 (10)	0.0517 (9)	0.0065 (7)	0.0101 (7)	0.0039 (7)
C10	0.0486 (8)	0.0493 (9)	0.0469 (9)	0.0009 (7)	0.0006 (7)	0.0010 (7)
C11	0.0541 (9)	0.0612 (11)	0.0534 (10)	0.0070 (8)	0.0056 (7)	0.0073 (8)
C12	0.0552 (9)	0.0707 (12)	0.0658 (11)	0.0123 (9)	0.0033 (8)	0.0006 (10)
C13	0.0575 (10)	0.0541 (10)	0.0645 (11)	0.0066 (8)	−0.0135 (8)	−0.0024 (9)
C14	0.0784 (12)	0.0683 (13)	0.0661 (12)	0.0075 (10)	−0.0006 (10)	0.0229 (10)
C15	0.0688 (11)	0.0702 (12)	0.0623 (11)	0.0096 (9)	0.0140 (9)	0.0164 (9)
C16	0.0582 (10)	0.0726 (13)	0.0632 (11)	0.0162 (9)	0.0233 (9)	−0.0002 (9)
C17	0.0782 (13)	0.0806 (16)	0.0993 (17)	0.0089 (12)	0.0215 (12)	−0.0109 (13)
C18	0.128 (2)	0.0684 (16)	0.124 (2)	0.0191 (17)	0.041 (2)	0.0056 (16)
C19	0.134 (3)	0.112 (3)	0.100 (2)	0.055 (2)	0.030 (2)	0.0151 (19)
C20	0.0854 (17)	0.151 (3)	0.099 (2)	0.042 (2)	−0.0056 (15)	0.000 (2)
C21	0.0668 (13)	0.0997 (18)	0.0919 (16)	0.0098 (12)	0.0120 (12)	−0.0023 (14)
C22	0.0885 (15)	0.0745 (15)	0.0986 (16)	0.0267 (12)	−0.0167 (13)	0.0054 (12)
N1	0.0523 (7)	0.0649 (10)	0.0581 (9)	0.0116 (7)	0.0170 (6)	0.0135 (7)
N2	0.0770 (11)	0.0546 (10)	0.1004 (14)	0.0011 (8)	0.0181 (10)	0.0176 (10)
O1	0.1073 (12)	0.1217 (14)	0.1512 (17)	0.0298 (11)	0.0902 (12)	0.0615 (13)
O2	0.1292 (15)	0.192 (2)	0.0477 (8)	0.0691 (15)	0.0026 (9)	−0.0158 (11)
O3	0.1342 (15)	0.0769 (11)	0.1182 (14)	−0.0176 (10)	0.0390 (11)	0.0365 (10)
O4	0.1345 (16)	0.1048 (15)	0.1263 (16)	−0.0521 (13)	−0.0165 (13)	0.0139 (13)
S1	0.0799 (3)	0.1018 (5)	0.0615 (3)	0.0312 (3)	0.0356 (3)	0.0208 (3)

### Geometric parameters (Å, °)

**Table:**

C1—C2	1.386 (3)	C13—C14	1.371 (3)
C1—C6	1.398 (2)	C13—C22	1.505 (3)
C1—N1	1.425 (2)	C14—C15	1.373 (3)
C2—C3	1.369 (4)	C14—H14	0.9300
C2—H2	0.9300	C15—H15	0.9300
C3—C4	1.373 (4)	C16—C17	1.373 (3)
C3—H3	0.9300	C16—C21	1.373 (3)
C4—C5	1.368 (4)	C16—S1	1.747 (2)
C4—H4	0.9300	C17—C18	1.394 (4)
C5—C6	1.389 (3)	C17—H17	0.9300
C5—H5	0.9300	C18—C19	1.330 (5)
C6—C7	1.443 (3)	C18—H18	0.9300
C7—C8	1.312 (3)	C19—C20	1.355 (5)
C7—H7	0.9300	C19—H19	0.9300
C8—N2	1.450 (3)	C20—C21	1.384 (4)
C8—C9	1.506 (2)	C20—H20	0.9300
C9—N1	1.471 (2)	C21—H21	0.9300
C9—C10	1.520 (2)	C22—H22A	0.9600
C9—H9	0.9800	C22—H22B	0.9600
C10—C11	1.372 (2)	C22—H22C	0.9600
C10—C15	1.382 (2)	N1—S1	1.6620 (14)
C11—C12	1.382 (2)	N2—O4	1.208 (2)
C11—H11	0.9300	N2—O3	1.214 (2)
C12—C13	1.378 (3)	O1—S1	1.4159 (19)
C12—H12	0.9300	O2—S1	1.428 (2)
			
C2—C1—C6	119.8 (2)	C13—C14—C15	122.10 (18)
C2—C1—N1	121.69 (19)	C13—C14—H14	119.0
C6—C1—N1	118.49 (16)	C15—C14—H14	119.0
C3—C2—C1	119.5 (2)	C14—C15—C10	120.53 (17)
C3—C2—H2	120.2	C14—C15—H15	119.7
C1—C2—H2	120.2	C10—C15—H15	119.7
C2—C3—C4	121.1 (2)	C17—C16—C21	120.3 (2)
C2—C3—H3	119.4	C17—C16—S1	120.10 (18)
C4—C3—H3	119.4	C21—C16—S1	119.64 (18)
C5—C4—C3	120.0 (3)	C16—C17—C18	119.0 (3)
C5—C4—H4	120.0	C16—C17—H17	120.5
C3—C4—H4	120.0	C18—C17—H17	120.5
C4—C5—C6	120.3 (3)	C19—C18—C17	119.8 (3)
C4—C5—H5	119.8	C19—C18—H18	120.1
C6—C5—H5	119.8	C17—C18—H18	120.1
C5—C6—C1	119.2 (2)	C18—C19—C20	122.3 (3)
C5—C6—C7	121.89 (19)	C18—C19—H19	118.8
C1—C6—C7	118.89 (18)	C20—C19—H19	118.8
C8—C7—C6	119.48 (16)	C19—C20—C21	119.1 (3)
C8—C7—H7	120.3	C19—C20—H20	120.5
C6—C7—H7	120.3	C21—C20—H20	120.5
C7—C8—N2	120.58 (17)	C16—C21—C20	119.5 (3)
C7—C8—C9	121.95 (16)	C16—C21—H21	120.2
N2—C8—C9	117.43 (18)	C20—C21—H21	120.2
N1—C9—C8	108.54 (14)	C13—C22—H22A	109.5
N1—C9—C10	109.71 (13)	C13—C22—H22B	109.5
C8—C9—C10	113.48 (13)	H22A—C22—H22B	109.5
N1—C9—H9	108.3	C13—C22—H22C	109.5
C8—C9—H9	108.3	H22A—C22—H22C	109.5
C10—C9—H9	108.3	H22B—C22—H22C	109.5
C11—C10—C15	117.95 (16)	C1—N1—C9	115.55 (13)
C11—C10—C9	122.75 (15)	C1—N1—S1	119.18 (11)
C15—C10—C9	119.24 (15)	C9—N1—S1	117.61 (13)
C10—C11—C12	120.91 (17)	O4—N2—O3	122.9 (2)
C10—C11—H11	119.5	O4—N2—C8	118.15 (19)
C12—C11—H11	119.5	O3—N2—C8	118.9 (2)
C13—C12—C11	121.32 (17)	O1—S1—O2	120.72 (13)
C13—C12—H12	119.3	O1—S1—N1	106.53 (10)
C11—C12—H12	119.3	O2—S1—N1	105.78 (9)
C14—C13—C12	117.16 (17)	O1—S1—C16	107.62 (11)
C14—C13—C22	121.08 (19)	O2—S1—C16	108.37 (12)
C12—C13—C22	121.75 (19)	N1—S1—C16	107.11 (8)
			
C6—C1—C2—C3	−0.1 (3)	C21—C16—C17—C18	0.4 (3)
N1—C1—C2—C3	−179.51 (17)	S1—C16—C17—C18	−179.70 (17)
C1—C2—C3—C4	0.5 (3)	C16—C17—C18—C19	0.6 (4)
C2—C3—C4—C5	−0.9 (4)	C17—C18—C19—C20	−0.4 (4)
C3—C4—C5—C6	0.9 (3)	C18—C19—C20—C21	−0.8 (5)
C4—C5—C6—C1	−0.6 (3)	C17—C16—C21—C20	−1.6 (3)
C4—C5—C6—C7	179.81 (19)	S1—C16—C21—C20	178.51 (18)
C2—C1—C6—C5	0.2 (3)	C19—C20—C21—C16	1.8 (4)
N1—C1—C6—C5	179.58 (15)	C2—C1—N1—C9	147.01 (16)
C2—C1—C6—C7	179.81 (15)	C6—C1—N1—C9	−32.4 (2)
N1—C1—C6—C7	−0.8 (2)	C2—C1—N1—S1	−64.2 (2)
C5—C6—C7—C8	−164.16 (17)	C6—C1—N1—S1	116.43 (15)
C1—C6—C7—C8	16.2 (2)	C8—C9—N1—C1	46.92 (18)
C6—C7—C8—N2	−179.99 (15)	C10—C9—N1—C1	−77.58 (17)
C6—C7—C8—C9	2.5 (2)	C8—C9—N1—S1	−102.38 (15)
C7—C8—C9—N1	−33.2 (2)	C10—C9—N1—S1	133.12 (12)
N2—C8—C9—N1	149.26 (15)	C7—C8—N2—O4	173.7 (2)
C7—C8—C9—C10	89.0 (2)	C9—C8—N2—O4	−8.7 (3)
N2—C8—C9—C10	−88.50 (19)	C7—C8—N2—O3	−7.0 (3)
N1—C9—C10—C11	133.39 (17)	C9—C8—N2—O3	170.59 (17)
C8—C9—C10—C11	11.8 (2)	C1—N1—S1—O1	48.87 (16)
N1—C9—C10—C15	−49.3 (2)	C9—N1—S1—O1	−162.97 (14)
C8—C9—C10—C15	−170.91 (17)	C1—N1—S1—O2	178.48 (15)
C15—C10—C11—C12	0.1 (3)	C9—N1—S1—O2	−33.35 (16)
C9—C10—C11—C12	177.40 (17)	C1—N1—S1—C16	−66.07 (15)
C10—C11—C12—C13	1.2 (3)	C9—N1—S1—C16	82.10 (14)
C11—C12—C13—C14	−1.3 (3)	C17—C16—S1—O1	159.82 (17)
C11—C12—C13—C22	177.92 (18)	C21—C16—S1—O1	−20.30 (19)
C12—C13—C14—C15	0.2 (3)	C17—C16—S1—O2	27.73 (18)
C22—C13—C14—C15	−179.0 (2)	C21—C16—S1—O2	−152.39 (16)
C13—C14—C15—C10	1.0 (3)	C17—C16—S1—N1	−85.98 (17)
C11—C10—C15—C14	−1.1 (3)	C21—C16—S1—N1	93.90 (16)
C9—C10—C15—C14	−178.56 (18)		

### Hydrogen-bond geometry (Å, °)

**Table:**

*D*—H···*A*	*D*—H	H···*A*	*D*···*A*	*D*—H···*A*
C4—H4···O4^i^	0.93	2.60	3.418 (4)	148

Symmetry codes: (i) *x*−1/2, −*y*+1/2, *z*−1/2.
